# The evaluation of breast findings detected through different visualisation techniques in acromegaly patients — a retrospective study

**DOI:** 10.3906/sag-2105-35

**Published:** 2021-09-16

**Authors:** Pınar AKHANLI, Sema HEPŞEN, Bekir UÇAN, Hakan DÜĞER, Hayri BOSTAN, Muhammed KIZILGÜL, Muhammed Erkam SENCAR, Erman ÇAKAL

**Affiliations:** Department of Endocrinology and Metabolism, University of Health Sciences, Dışkapı Yıldırım Beyazıt Training and Research Hospital, Ankara, Turkey

**Keywords:** Acromegaly, breast density, breast neoplasm, breast cancer

## Abstract

**Background/aim:**

It is known that the increased growth hormone (GH) and insulin-like growth factor-1 (IGF-1) have mitogenic and antiapoptotic properties in breast cells in acromegaly. Our study aims to evaluate breast findings in patients with acromegaly by comparing them to the control group.

**Materials and methods:**

Sixty-one patients followed with acromegaly diagnosis and 180 healthy controls were included in our study. Demographic data, laboratory results, Breast Imaging-Reporting and Data System (BI-RADS) scores, and breast density evaluated via mammography, malign and benign breast lesions evaluated via mammography, breast ultrasonography (USG), and breast magnetic resonance imaging (MRI) of patients were compared to the control group.

**Results:**

While BI-RADS scores were similar in patient and control groups, breast density in acromegaly patients was found out to be higher compared to the control group (*p =* 0.754, *p =* 0.001, respectively). In acromegaly patients, the breast calcification rate was higher than controls (*p =* 0.021). t was observed that mass frequency in USG in acromegaly patients increased when GH level increased as well (*p =* 0.021). No difference was detected between benign and malign breast lesions diagnosed histopathologically (*p =* 0.031, *p =* 0.573, respectively). There was not any difference in terms of BI-RADS scores, breast types, and breast lesions in acromegaly patients that were in remission and not in remission (*p >* 0.05).

**Conclusion:**

Benign and malign breast lesions were found out to be similar to the control group, although breast density rate was detected to be higher in acromegaly patients. A regular follow-up is required in these patients via suitable breast visualization techniques considering their age and clinical status due to mass formation risk derived from increased GH level and extreme breast density despite the absence of any detected breast lesion frequency in acromegaly patients.

## 1. Introduction

Acromegaly is a rare disorder, which progresses depending on an increase in GH secretion and is derived from a pituitary adenoma in general [1]. The estimated incidence is nearly 4 cases per million/year in the general world population and the prevalence 85 per million [2]. It is reported that the mean age of diagnosis for acromegaly is between 40 and 50 [3]. When growth hormone (GH) secretion is high, it stimulates the hepatic secretion of insulin-like growth factor-1 (IGF-1), which causes the majority of acromegaly clinical symptoms [4]. Patients can apply to a hospital with comorbidities related to extreme GH or IGF-1 levels such as diabetes or glucose intolerance, hypertension, obstructive sleep apnea syndrome, cardiomyopathy, and goiter [3]. An increase in morbidity and mortality rates depending upon cardiovascular, cerebrovascular, and metabolic complications secondary to acromegaly has been shown. It is considered that high GH and IGF-1 levels can lead to cancer incidence increase in acromegaly patients due to mitogenic and antiapoptotic properties [5,6]. The increased risk of benign and malignant tumors in acromegaly patients keeps on being a matter of debate. Ultrasonography (USG), mammography, and magnetic resonance imaging (MRI), when required, are used to detect breast lesions in general. Breast density is also evaluated via mammography in addition to lesions. Immense breast tissue in mammography can affect the risk of breast cancer. Although breast lesions are generally benign, they can also be precancerous and malignant. Breast cancer is the most common cancer type seen in females and comes after lung cancer in cancer mortality [7]. Several research revealed that GH has an important role on breast cell oncogenic transformation and progression as both in vitro and in vivo [8]. While an association between acromegaly and breast cancer in several studies is revealed [2,9], this association cannot be indicated in some others [10,11].

Our study aims to compare benign and malign breast lesions, Breast Imaging-Reporting and Data System (BI-RADS) scores, and breast density in acromegaly patients retrospectively with control group through mammography, USG, and MRI.

## 2. Material and methods

### 2.1. Study design

This study was designed as a retrospective study. The Ethics Committee of our institute approved this study regarding the principles of the Declaration of Helsinki. Written informed consent was taken from subjects before taking part in the study.

### 2.2. Patients and laboratory tests

Sixty-one female patients diagnosed with acromegaly were followed and treated in Ankara Dışkapı Training and Research Hospital Endocrinology and Metabolic Diseases Department between 2008 and 2019 were compared to 180 female controls who accept to be part of our study. Acromegaly diagnosis and active disease definition were determined utilizing clinical findings, GH levels, GH suppression below 1 g/L during oral glucose tolerance test (OGTT), increased IGF-1 levels regulated according to age and sex, and follow-up findings after surgery [9]. IGF-1 and GH values of acromegaly patients measured in 3 months before breast imagings of patients were recorded. Multiple endocrine neoplasia (MEN) patients were excluded from the study. The information related to chronic diseases of patients was obtained from hospital records. Demographic features of patients, laboratory test results, mammography, breast USG, and MRI results were recorded. BI-RADS scores and breast densities determined by mammography (A, B, C, D according to BI-RADS density classification) [10], benign calcification, macrocalcification, and microcalcification presence, asymmetric density, intramammary lymph node, and images that resemble a mass as well as BI-RADS scores determined by only MRI were also recorded. Mammography was based on for BI-RADS scores in patients having both mammography and MRI. Fibrocystic breast pattern, ductal ectasia, fibroadenoma, apse, images compatible with hamartoma, cystic and solid lesions determined by USG were recorded in detail. Biopsy results of recorded lesions, if they were carried out, were recorded. The malign lesion diagnosis of patients were based on histopathologic diagnosis.

Control group was composed by scanning retrospective breast workups belonging to routine breast examinations performed in Ankara Dışkapı Training and Research Hospital Internal Diseases Department between 2018 and 2019. Consents of the controls were taken by calling them. More than one doctor in the mammography unit in our clinic evaluated radiological findings. We evaluated the results of the patients retrospectively through the system. The first workouts of patients were based on. Those patients who are followed-up for any breast pathology were excluded from the study.

### 2.3. Statistical analysis

Statistical analyses were performed using the SPSS software version 21 (Chicago, IL). The variables were investigated through visual (histograms, probability plots) and analytic methods (Kolmogorov–Smirnov/Shapiro–Wilk’s test) to determine whether they were normally distributed or not. While the Student’s t-test was used to compare tumor size detected with USG, the Mann–Whitney U test was performed to compare other variables between groups. Categorical data were presented as numbers and percentages (%). Descriptive analyses were presented using means and standard deviations for normally distributed variables, whereas medians and interquartile ranges (IQR) are at the 25th and 75th percentiles for non-normally distributed variables. The proportions of mammography and USG findings of patients in remission or not were presented by using cross-tabulations. The Chi-square test or Fisher’s exact test, where appropriate, was used to compare these proportions. A p-value, less than 0.05 was considered to show a statistically significant difference. While investigating the associations between breast findings and other variables, correlation coefficients and their significance were calculated using the Spearman test.

## 3. Results

Sixty-one female acromegaly patients and 180 female controls were included in the study. Median ages of patients and controls were 53 (IQR 25–75; 45–59) and 48 (IQR 25–75; 41–55), respectively. Total disease duration of acromegaly patients was 7 (IQR 25–75; 2.5–11) years. While median IGF-1 level of patients at the diagnosis time was 655 (IQR 25–75; 528–901) ng/mL, median GH level was 6.3 (IQR 25–75; 3.6–10) ng/mL. IGF-1 and GH values of the patients measured on the closest date to the period at which imaging methods were performed were 201 (IQR 25–75; 116.5–321.5) and 0.8 (IQR 25–75; 0.14–2.21), respectively. Calculated IGF-1 values according to age and gender at the diagnosis time of 53 (86.9%) patients were higher than 97th percentile, 7 (11.5%) were between 90th and 95th percentile, and 1 (1.6%) was between 90th and 95th percentile. There were macroadenoma in 48 (78.7%) patients and microadenoma in 13 (21.3%) patients. The mean tumor size was 14.5 (11–20) mm. Hypertension (37.7%), diabetes mellitus (34.4%), and coronary artery disease (6.6%) were additional diseases in the acromegaly group. TN/TS surgery was performed on 54 (88.5%) patients as an initial treatment. The number of patients who received an additional treatment after surgery was as follows: 20 (32.8 %) patients-octreotide, 12 (19.7%) patients-lanreotide, 6 (9.8%) patients-cabergoline in addition to somatostatin analog treatment, 1 (1.6%) patient-pegvisomant treatments. In addition to these data, radiotherapy and gamma knife were performed on 2 (3.3%) and 5 (8.2%) patients, respectively, as an additional treatment. Table 1 shows baseline characteristics, treatment details, and laboratory test results of the subjects.

Considering the techniques performed on the patients in the acromegaly group and on the subjects in the control group, the data were as follows respectively: 32 (52.5%) patients and 43 (23.9%) subjects-mammography, 10 (16.4%) patients and 43 (23.9%) subjects-USG, 1 (1.6%) patient and 4 (2.2%) subjects-MRI, and 18 (29.5%) patients and 87 (48.3%) subjects-both USG and mammography. Moreover, in the control group, 1 subject (0.6%) was performed both mammography and MRI, 1 subject (0.6%) was performed both USG and MRI, and 1 subject (0.6%) was performed USG, MRI, and mammography.

BI-RADS scores were similar in the patient and control groups (*p =* 0.580). A significant difference was detected among breast types showed by mammography between the acromegaly and control groups (*p =* 0.001). Calcification presence was detected in 45 (90%) acromegaly patients and 101 (74.3%) subjects in the control group. Calcification rate was higher in acromegaly patients than control group (*p =* 0.021). The number of benign calcification, microcalcification, and macrocalcification was 38 (84.5%), 6 (13.3%), and 1 (2.2%) in the acromegaly group and 91 (90.1%), 10 (9.9%), and 0 (0%) in the control group, respectively. The presence of asymmetric density was similar in the patient and control groups (*p =* 0.423). The presence of intramammary lymph node in mammography was also similar in both groups (*p =* 0.276). There was no difference in mass frequency detected in mammography between the two groups (*p =* 0.187). Cystic and solid masses were 0 (0%) and 2 (100%) in acromegaly patients whereas 7 (38.9%) and 11 (61.1%) in the control group, respectively. Similarly, there was no difference in mass frequency detected in USG in both groups (*p =* 0.103). While cystic and solid masses detected in USG in acromegaly patients were 7 (70%) and 3 (30%) respectively, and they were 39 (66.1%) and 20 (11.1%) in the control group. The presence of ductal ectasia and fibrocystic breast were similar in both groups (*p =* 0.223, *p =* 0.226; respectively). The presence of intramammary lymph nodes in USG was also similar in two groups (*p =* 797). Table 2 and 3 show breast lesions of the patients detected via mammography and USG, respectively. Breast cancer frequency detected in both groups was similar (*p =* 0.573). There was no patient with any benign breast lesions in the acromegaly patients, however, there were 13 (7.2%) patients in the control group (*p =* 0.031). In the acromegaly group, 2 (66.7%) patients were with invasive ductal and 1 (33.3%) patient was with mucinosis breast cancer. In the control group, on the other hand, 1 (16.7%) patient was with ductal carcinoma in situ (DCIS), 1 (16.7%) patient was with lobular carsinoma in situ (LCIS), and 4 patients (66.7%) were with invasive ductal carcinoma. Any acromegaly patient as not diagnosed with breast cancer before acromegaly diagnosis. Details belonging to histopathological findings of malign and benign breast lesions are shown in Table 4. There was no detected lesion in MRI in the patient group, however, 1 patient was detected with two solid lesions on the right and left (the biggest one was 11 mm) in the control group.

When acromegaly patients were evaluated, no correlation was observed between BI-RADS scores and breast density and age, disease duration, GH, and IGF-1 levels (*p >* 0.05 for each). It was observed that mass frequency in USG in acromegaly patients increased when GH level increased as well (*p =* 0.021). The presence of fibrocyst decreased acromegaly patients depending upon age increase (*p =* 0.018, *p =* 0.072, respectively). There was no correlation between calcification, intramammary lymph node, ductal ectasia, mass presence detected in mammography and breast cancer, and age, disease duration, GH, and IGF-1 levels (*p >* 0.05 for each). 40 (65.6%) patients were in remission in the period of workup, however 21 (34.4%) patients were not. Median IGF-1 and GH in the period of workup were 201 (IQR 25–75; 118–323) and (IQR 25–75; 0.14–2.16) ng/mL, respectively. BI-RADS scores and breast types were similar in patients in remission and not (*p =* 0.527, *p =* 0.754, respectively). Median BI-RADS of patients in remission was observed as 2 (IQR 25–75; 2–2), and this rate was 2 (IQR 25–75; 1.25–2) for patients not in remission. A mass was detected through mammography in 1 (2.8%) and 2 (16.7 %) patients who were in remission and not, respectively (*p =* 0.089). There was no difference in asymmetric density rate between patients in remission and not (*p =* 0.490). The number of the patients being in remission and not in remission and detected calcification through mammography was 36 (94.7%) and 9 (75%), respectively (*p =* 0.049).

The presence of mass detected through USG was observed in 6 (28.6%) patients in remission and in 4 (26.7%) patients not in remission (*p =* 0.924). There was also no difference in fibrocyst and ductal ectasia rate detected through USG between the patients who were in remission and not in remission *(p =* 0.614, *p =* 0.313, respectively). Breast cancer was detected in 1 (2.5%) and 2 (9.5%) patients being in remission and not, respectively.

## 4. Discussion

This study revealed that there was no difference in benign and malign breast lesions between acromegaly patients and the control group. When compared acromegaly patients in remission to the patients who were not in remission, similarly, no difference was found out. Besides, breast density in acromegaly patients was detected to be higher than the control group. It was also revealed when the GH level increased, mass frequency in USG increased.

It is difficult to analyse breast lesions in acromegaly patients due to the fact that acromegaly is a rare disease. Extreme breast density in mammography can affect breast cancer risk. Extreme tissue can make detecting small lesions difficult by reducing the sensitivity of mammography [11,12]. In addition to this, increased density is an independent risk factor of breast cancer because the vast majority of cancer progresses in glandular parenchyma [13]. Breast density in mammography can show a change according to the operator [14]. Mammographic density is not related to breast firmness or breast size [15]. Breast density is higher in young women and differs according to genetic factors, estrogen using, climacteric, parity, and tamoxifen using [16].

In our study BI-RADS scores were similar the patient and control groups, however, breast density showed via mammography was significantly high in acromegaly group. We did not detect any correlation between BI-RADS scores, breast density and age, disease duration, GH, and IGF-1 levels.

According to a report published by Tacliafico et al., BI-RADS scores and breast density of females with acromegaly were significantly high when 30 premenopausal patients with acromegaly compared to 60 premenopausal controls. A positive correlation was found out between IGF-1 levels and disease duration and mammographic breast density [17].

In contrast with this study, an association between IGF-1, seven subgroups of IGFBP and volumetric density measures and area density measures was researched in a study conducted by Hada et al with 293 females between 40 and 45 years. While new positive correlations between IGFBP-2 and breast density percentage were detected, no positive correlation was detected between IGF-1, IGFBP3 and breast density [18]. No postmenopausal patient to be included in our study could be the reason why BI-RADS scores are similar with the control group and why there is no correlation among GH, IGF-1 levels and disease duration, age factor, and mammographic breast density.

As far as we know, there are many studies evaluating malign breast lesions in females with acromegaly, however there are not any studies evaluating benign breast lesions in literature. Up to now, there has not been a detailed study analyzing both benign and malign breast lesions in literature, without our study.

We revealed that calcification rate was significantly higher in females with acromegaly than the control group when breast lesions were evaluated.

According to a report published by Stompel et al, with 29% of females between 45–49, 34% between 50–54, and 43% between 55–59 years were with benign calcification [16]. In our study, benign calcification rate of acromegaly patients was 84%, which is higher than studies in literature. In addition to that, we did not detect increased benign and malign lesion frequency in the patient group compared to the control group. Asymmetric density detected via mammography, fibrocystic disease and ductal ectasia detected via USG, intramammary lymph node detected via both mammography and USG were similar in both groups. In literature, there is no clear datum related to the prevalence of breast lesions in acromegaly patients.

The upper limit of normal IGF-1 is associated with high breast, prostate, colorectal, and lung cancer risk in general population [4]. GH affects mammary gland growth and lactation period. It shows this effect with not only by itself but also with estrogen and progesterone. The effect of GH on breast cancer cells can be through IGF-1 or proliferative effect independent from IGF-1. Higher GHR, IGF-1, and IGF-1R expression are observed on breast cancer cells in humans. GH tumor expression is associated with metastatic breast cancer and poor prognosis positively [19]. The proportion of malign lesion in the patient group was not found out to be higher than the control group. Comorbidity rates of the patient and control groups were similar. For this reason, we consider that the effects of chronic diseases such as diabetes for which it is possible to see frequent malignity rates can be similar. We observed that mass frequency increased depending upon an increase in the GH level.

There are a large number of studies analysing malign breast lesions in acromegaly patients. Within these studies, a control group is included in two studies similar to our study. These control groups are constituted of patients with nonfunctional adenoma and prolactinoma.

Popovic et al. compared patients with acromegaly, nonfunctional adenoma, and prolactinoma in their published case-control study. Acromegaly patients had a 3.39-fold increased rate of malignity in general population. The rate of breast cancer incidence in acromegaly group was found to be higher compared to the control group; however, the difference was not statistically significant [20]. In another case-control study published by Wolinski et al., it was revealed that breast cancer is frequently seen in the acromegaly group than the control group consisted of patients with nonfunctional adenoma and prolactinoma [21]. The association between acromegaly and breast cancer frequency was not revealed clearly in studies analysing only acromegaly patients.

In another study, no correlation could be found out between IGF-1, GH levels and cancer in 445 acromegaly patients. Furthermore, no increase has been found out in breast cancer; however a poor correlation has been detected between thyroid cancer frequency and acromegaly. It is suggested that if studies correlated with high IGF-1, GH levels, and high cancer incidence are conducted through high-sensitive measures of today’s technology, lower standardized incidence rate is detected [22]. In a retrospective study analysing cancer incidence and mortality in which 1362 acromegaly patients were included, no increase in breast cancer was observed. In addition to this, it was indicated that there was no increase in mortality rate in malignant diseases, but colon cancer mortality rate was higher than expected [23]. On the other hand, Nabarro et al. revealed that there was a fourfold increase in breast cancer incidence in acromegalic patients [24]. An another study showed that there was a slight increase in all cancer types including breast cancer [2].

An analysis of UK Biobank data showed that high IGF-1 concentration in premenopausal and postmenopausal females is associated with breast cancer risk increase [25]. Guides for acromegaly patients’ management do not include any scanning for breast tumors. Our data are consistent with the instructions of the guides.

We compared our data to studies conducted in Turkey in order to equalize ethnic and social factors in breast cancer incidence in acromegaly patients. According to Turkey cancer statistic 2015, the most common cancer type is breast cancer in females in Turkey. (43,8/100.000 per person, Age Standardized Rate) According to a study published by Dağdelen et al., breast cancer rate is 2.5% in 160 acromegaly patients [26] while this rate is 2.8% for 104 acromegaly patients with regard to the study conducted by Güllü et al [27]. We detected breast cancer rate as 3.3% in acromegaly patients and this rate was similar to previous studies in Turkey.

Compared to acromegaly patients among themselves, how patients in remission or not in remission have an effect on the presence of breast lesions has not been proved.

As you realize, there are no study in literature comparing benign breast lesions between acromegaly patients in remission and not in remission. Güllü et al. revealed that remission duration is significantly longer in acromegaly patients with cancer diagnosis. No additional information has been found out on breast cancer [27].

The strengths of our study are that all breast lesions have not been evaluated in detail and our study has a remarkable amount of controls. That our study is a retrospective study and that data cannot be generalized to acromegaly patients in the whole ethnic population are the limitations of our study. Another limitation is that radiology findings have not been evaluated by a single expert because of the retrospective design.

As a consequence, according to our results, benign and malign breast lesions in acromegaly patients were similar to controls. Also, remission status of the patients appeared not to have an effect on the progression of lesions. In addition to them, we detected that breast density was higher in acromegaly patients than the control group and mass frequency increased depending upon an increase in the GH level.

It should be kept in mind that increased breast density and mass formation in breast increase breast cancer risk. For this reason, patients should be regularly followed up through suitable breast visualization techniques by taking into consideration their age and clinical status.

## Figures and Tables

**Table 1:** Baseline characteristics, treatment details, and laboratory test results of the subjects.

	Acromegaly patients	Controls	*p* value
**Demographic data and laboratory test results**			
**Number, n**	61	180	
**Age, years**	53 (45–59)	48 (41–55)	0.067
**Active disease, n (%)**	21 (34.4%)		
**Total disease duration, years**	7 (2.5–11)	-	
**Macroadenoma, n (%)**	48 (78.7)		
**Tumor size, mm**	14.5 (11–20)		
**IGF-1, baseline (ng/mL)**	655 (528–901)	-	
**GH, baseline (ng/mL)**	6.3 (3.6–10)	-	
**IGF-1, measured on the closest date to the visualisation techniques performed, (ng/mL)**	201 (116.5–321.5)		
**GH, measured on the closest date to the visualisation techniques performed, (ng/mL)**	0.8 (0.14–2.21)		
**Treatment**			
**Surgery, n (%)**	54 (88.5)		
**Octreotide, n (%)**	20 (32.8)		
**Lanreotide, n (%)**	12 (19.7)		
**Pegvisomant, n (%)**	1 (1.6)		
**Cabergoline, n (%)**	6 (9.8)		
**Gama knife, n (%)**	5 (8.2)		
**Radiotheraphy, n (%)**	2 (3.3)		
**Comorbidities**			
**Hypertension, n (%)**	23 (37.7)	42 (23.3)	**0.029**
**Diabetes mellitus, n (%)**	21 (34.4)	43 (23.9)	0.108
**Heart failure, n (%)**	0 (0)	2 (1.1)	0.409
**CAD, n (%)**	4 (6.6)	5 (2.8)	0.179

IGF-1: Insulin-like growth factor, GH: Growth hormone, CAD: Coronary artery disease.

**Table 2:** Breast lesions in mamography of the subjects

	Acromegaly patients	Controls	*p* value
**BIRADS Score**	2 (2–2)	2(2–2)	0.754
**BIRADS 0 Score, n (%)**	3 (6)	8 (5.9)	
**BIRADS 1 Score, n (%)**	3 (6)	13 (9.6)	
**BIRADS 2 Score, n (%)**	36 (72)	84 (61.8)	
**BIRADS 3 Score, n (%)**	5 (10)	25 (18.4)	
**BIRADS 4 Score, n (%)**	1 (2)	4 (2.9)	
**BIRADS 5 Score, n (%)**	2 (4)	2 (1.5)	
**Breast density**			**0.001**
**A type, n (%)**	2 (4)	16 (11.8)	
**B type, n (%)**	18 (36)	74 (54.4)	
**C type, n (%)**	29 (58)	45 (33.1)	
**D type, n (%)**	1 (2)	1 (0.7)	
**Calcifications, n (%)**	45 (90)	101 (74.3)	**0.021**
**Benign, n (%)**	38 (84.5)	91 (90.1)	
**Micro, n (%)**	6 (13.3)	10 (9.9)	
**Macro, n (%)**	1 (2.2)	0 (0)	
**Asymetric density, n (%)**	21 (42)	40 (35.4)	0.423
**Breast lesions**	3 (6.3)	18 (13.4)	0.187
**Cystic, n (%)**	0 (0)	7 (38.9)	
**Solid, n (%)**	3 (100)	11 (61.1)	
**Tumor size, mm**	20 ± 8	19.4 ± 6.1	0.878
**IMLN**	5 (9.1)	20 (15)	0.276

BIRADS: Breast Imaging-Reporting and Data, IMLN: Intramammary lymph node.

**Table 3:** Breast lesions in ultrasonography of the subjects.

	Acromegaly patients (n = 61)	Controls (n = 180)	*p* value
**Patients with breast ultrasonography, n (%)**	49 (81.7)	132 (73.3)	
**Breast lesions, n (%)**	10 (27.8)	59 (42.8)	0.103
**Cystic, n (%)**	7 (70)	39 (66.1)	
**Solid, n (%)**	3 (30)	20 (11.1)	
**Tumor size, mm**	10.5 (4.7–15.5)	10 (5–18)	0.925
**Ductal ectasia, n (%)**	2 (3.3)	14 (7.9)	0.223
**Fibrocystic breast, n (%)**	2 (3.3)	14 (7.9)	0.226
**IMLN, n (%)**	3 (6.8)	8 (5.8)	0.797

IMLN: Intramammary lymph node.

**Table 4:** Details belonging to histopathological findings of malign and benign breast lesions.

	Acromegaly patients	Controls	*p* value
**Malign, n (%)**	3 (4.9)	6 (3.3)	0.573
**DCIS, n (%)**	0 (0)	1 (16.7)	
**LCIS, n (%)**	0 (0)	1 (16.7)	
**İ** **nvasive ductal carcinoma, n (%)**	2 (66.7)	4 (66.7)	
**İ** **nvasive lobular carcinoma, n (%)**	0 (0)	0 (0)	
**Mucinous carcinoma, n (%)**	1 (33.3)	0 (0)	
**Benign, n (%)**	0 (0)	13 (7.2)	**0.031**
**Fibroadenoma, n (%)**	0 (0)	38.5	
**Hamartoma, n (%)**	0 (0)	15.4	
**Abscess, n (%)**	0 (0)	15.4	
**Lipoma, n (%)**	0 (0)	30.8	

DCIS: ductal carcinoma in situ LCIS: lobular carcinoma in situ.

## Data Availability

The datasets used and/or analyzed during the current study available from the corresponding author on reasonable request.
